# Quantitative Analysis of Elements in Fertilizer Using Laser-Induced Breakdown Spectroscopy Coupled with Support Vector Regression Model

**DOI:** 10.3390/s19153277

**Published:** 2019-07-25

**Authors:** Wen Sha, Jiangtao Li, Wubing Xiao, Pengpeng Ling, Cuiping Lu

**Affiliations:** 1 Key Laboratory of Intelligent Computing and Signal Processing of Ministry of Education, School of Electric Engineering and Automation, Anhui University, Hefei 230061, China; 2 Laboratory of Intelligent Decision, Institute of Intelligent Machines, Chinese Academy of Sciences, Hefei 230031, China

**Keywords:** fertilizer, support vector regression, laser-induced breakdown spectroscopy, grid method, genetic algorithm, particle swarm optimization, least squares

## Abstract

The rapid detection of the elements nitrogen (N), phosphorus (P), and potassium (K) is beneficial to the control of the compound fertilizer production process, and it is of great significance in the fertilizer industry. The aim of this work was to compare the detection ability of laser-induced breakdown spectroscopy (LIBS) coupled with support vector regression (SVR) and obtain an accurate and reliable method for the rapid detection of all three elements. A total of 58 fertilizer samples were provided by Anhui Huilong Group. The collection of samples was divided into a calibration set (43 samples) and a prediction set (15 samples) by the Kennard–Stone (KS) method. Four different parameter optimization methods were used to construct the SVR calibration models by element concentration and the intensity of characteristic line variables, namely the traditional grid search method (GSM), genetic algorithm (GA), particle swarm optimization (PSO), and least squares (LS). The training time, determination coefficient, and the root-mean-square error for all parameter optimization methods were analyzed. The results indicated that the LIBS technique coupled with the least squares–support vector regression (LS-SVR) method could be a reliable and accurate method in the quantitative determination of N, P, and K elements in complex matrix like compound fertilizers.

## 1. Introduction

The foundation of precise fertilization is accurately obtaining the content of elements in compound fertilizers to maximize their benefits. At present, the main sensing methods used by fertilizer manufacturers are national standard methods [1], inductively coupled plasma–atomic emission spectroscopy (ICP-AES) [2], flame atomic absorption spectrometry (FAAS) [3], atomic absorption spectroscopy (AAS) [4], near infrared reflectance spectroscopy (NIRS) [5], etc. These sensing methods need field samples and pretreatment before laboratory analysis, which are time-consuming, labor-intensive, and expensive requirements. Meanwhile, due to the contents of N, P, and K in compound fertilizer being typically high, fertilizer must be diluted several times before measuring, which leads to increase systematic errors. Further, these sensing methods cannot provide real-time information during the production process. At present, the pass rate of compound fertilizer products is only 97% [6], and non-qualifying products need to be returned to the factory for reprocessing, causing huge economic losses. Therefore, a technology that can quickly and accurately detect the elemental content in compound fertilizers is urgently needed.

Laser-induced breakdown spectroscopy (LIBS) is an ideal laser high-temperature ablation spectroscopy technique because it provides fast (in the order of milliseconds), insitu (smaller sample size), non-destructive, safe, environmentally friendly (no secondary pollution), multi-element analysis, and direct analysis of any state of matter. It has been successfully used in applications relating to water pollution [7], coal combustion [8], agriculture [9], space exploration [10], etc. In recent years, some studies have used the LIBS technique to detect the components of fertilizer. Andrade et al. turned liquid fertilizer into a solid state and detected its content using the LIBS technique. The levels of Cu, K, Mg, Mn, Zn, As, Cd, Cr and Pb in the fertilizer were analyzed, and the detection error range was ~0.02%–0.06%, which demonstrated that this method provides accurate measurement of liquid fertilizer [11]. Nicolodelli et al. used single-pulse and dual-pulse LIBS technology to measure phosphate rock and organic phosphate fertilizer. The samples were identified by principal component analysis (PCA) and partial least squares regression (PLSR), and the recognition result was able to reach a 95% confidence level, which showed that LIBS technology can be used to rapidly classify phosphate fertilizers in situ [12]. Yao et al. analyzed phosphorus and potassium elements in compound fertilizers. A quantitative analysis model was established by using the partial least squares (PLS) method in Unscrambler software. The obtained results were superior to those obtained using traditional methods, but the accuracy of detection still needed improvement [13]. Marangoni et al. used LIBS technology to analyze phosphorus in 26 different organic and inorganic fertilizers. Baseline correction and peak intensity normalization were used to pre-process the spectrum. The correlation between the measured value of LIBS and the true value was improved, but the absolute error of the two verification samples was close to 5% [14]. Andradeet al. optimized the LIBS system parameters and directly analyzed the levels of Cd, Cr, Pb, B, Cu, Mn, Na, Zn, Ca, and Mg elements in solid compound fertilizer. The quantitative analysis results were compared to those obtained using ICP-AES, and the correlation was good. The detection limit of the above elements was determined to be ~2 ppm–1%. These results demonstrated the ability of LIBS to be used for rapid analysis of fertilizer [15]. Liao et al. used LIBS to analyze the phosphorus content in compound fertilizer. The correlation coefficient increased from 0.83 to 0.98 when considering the influence of the oxygen characteristic line, and the relative error was only ~0.38%–1.70%. However, the number of samples was too small, so further modeling should be completed if the method is to be applied to actual field detection [16].

The above studies all showed that LIBS technology can be used to detect the types and contents of elements in chemical fertilizers. However, the accuracy and reliability of the quantitative analysisis still a shortcoming of LIBS, which greatly limits its practical application. Thus, there are many problems to be solved before it can be used in actual applications in rapid sensing.

Support vector regression (SVR) is a machine learning method based on statistical learning theory. It uses interval maximization to carry out model training by mapping difficult problems in the original space to a higher-dimensional space and seeking interval maximization to calculate the optimal linear hyper plane. The number of compound fertilizer samples collected in this experimental method was small, which is suitable for the statistical analysis of small samples [17]. In small samples, nonlinear pattern recognition has certain advantages. Zhang et al. employed a LIBS technique coupled with SVR and PLS methods to perform quantitative and classification analysis of 20 slag samples. The results showed that the SVR model could eliminate the influence of nonlinear factors due to self-absorption in the plasma and provide a better predictive result. It has been confirmed that the LIBS technique coupled with the SVR method is a promising approach to achieving the online analysis and process control of slag [18]. Shi et al. compared PLSR and SVR methods for quantitative analysis of the concentrations of five main elements (Si, Ca, Mg, Fe, and Al) in sedimentary rock samples. The parameter optimization method used was genetic algorithm (GA). The results demonstrated that the SVR model performed better, with more satisfactory accuracy and precision under the optimized conditions [19]. He et al. employed single-pulse and double-pulse LIBS to analyze nutrient elements in soil. Good performance was obtained using the PLSR and LS-SVR calibration model, with R^2^ greater than 0.95 in both the calibration and prediction sets for all nutrient elements. The results indicated that LIBS combined with PLSR and LS-SVR could be a good method for detecting nutrient elements in soil [20]. Liu et al. also used the PLSR and LS-SVR quantitative analysis method to detect the Cd content in soil. The results showed that the LS-SVR model under an Ar atmosphere obtained the best performance. The root-mean-square error for calibration (RMSEC) and the root-mean square error for prediction (RMSEP) were only 0.026 and 0.034, respectively, which demonstrated the ability of LIBS for the accurate quantitative detection of Cd in soil [21]. To the best of our knowledge, the simultaneous quantitative detection of N, P, and K elements based on LIBS coupled with SVR models by different parameter optimization methods has not been investigated.

The purpose of this paper was to explore the detection ability of LIBS for N, P, and K elements in fertilizer, and to find a fast and accurate quantitative analysis method. A selection of 58 fertilizer samples were provided by Huilong Chemical Fertilizer Plant, Anhui, China. The contents of all three elements in compound fertilizer were determined by ICP-AES. Four different parameter optimization methods were employed to establish the SVR model for quantitative analysis of all elements. The accuracy levels of the four SVR models were compared based on the performance of each model.

## 2. Materials and Methods

### 2.1. Sample Preparation

In this study, 58 compound fertilizer samples were collected and placed into sealed plastic bags to avoid contamination. As the compound fertilizer products had been pelletized, they had to be broken into powder before spectral scanning. All fertilizer powders were then sieved through a 60 mesh screen. A total 2 g of powder from each of the 58 fertilizer samples was weighed and pressed into tablets 30 mm in diameter and 2 mm in thickness, using 5 MPa force for 60 s (769YP-40C, KQ, Tianjin, China). The reference concentrations of N, P, and K in these samples were analyzed by ICP-AES. The statistics of the N, P, and K concentrations in the compound fertilizer samples are listed in Table 1.

### 2.2. Experimental Setup

**Figure 1:**
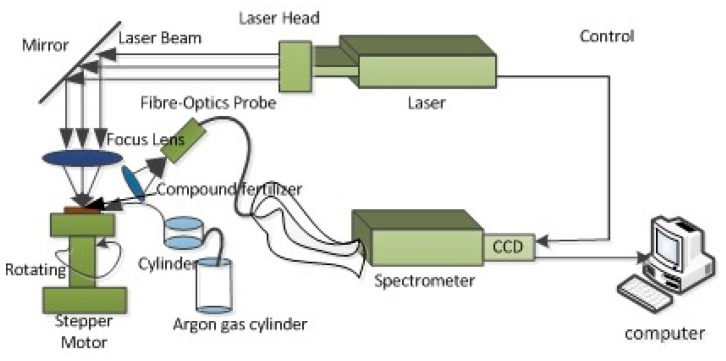
Schematic diagram of the laser-induced breakdown spectroscopy (LIBS) system for fertilizer samples.

The self-built LIBS system used in this experiment is shown in Figure 1. A Q-switched Nd: YAG pulsed laser (ICE450, 1064 nm, 6 ns pulse duration, Big Sky Laser Technologies, Morgan Hill, CA, USA; note that the company has changed its name to Quantel Laser) was used to generate the plasma on the compound fertilizer pellet. The pulse laser energy was 100 mJ, and it was focused with a 2.54 cm diameter, 4.5 cm focal length convex lens onto the fertilizer sample. The spot diameter size of the pulsed laser was approximately 0.5 mm, and the peak power density on the compound fertilizer sample was able to reach 2.2 GW/cm^2^. The light emitted from the plasma was collected via a quartz lens with 3.5 cm focal length and transmitted via an optical fiber with a diameter of 200 μm to a spectrometer (Avantes-ULS2048-USB2, Avantes, Apeldoorn, The Netherlands). The spectrometer used has four channels containing separate gratings and a charge-coupled device array, and all spectra were taken simultaneously in the wavelength ranges of 190–510 and 690–890 nm. The resolution of the spectrometer was approximately 0.1 nm. The spectrometer was triggered by the laser Q-switch output, and it had a digital delay generator which can control the gate delay. In this experiment, the delay time and the integration time were set for spectra acquisition at 1.28 μs and 1.05 ms (spectrometer minimum integration time), respectively [22]. Sample tablets were placed on the X-Y rotary stage, the speed of which can be adjusted by stepper motor, and the laser beam was adjusted to focus 3 mm below the sample surface to acquire LIBS spectra [23]. Argon was then passed through the cylinder, draining the air and forming an Ar atmosphere on the surface of the compound fertilizer sample, as shown in Figure 1. Thus, the sample was immersed in an Ar atmosphere. In order to eliminate shot-to-shot fluctuation, each sample was measured eight times, and each spectrum was collected with an average of 20 laser shots. 

### 2.3. SVR Algorithm Model Establishment

Under local thermal equilibrium (LTE) conditions and ignoring self-absorption effects, the measured characteristic atomic spectrum of ionic line intensity in LIBS spectroscopy can be expressed as

(1) $$I_{k,i}=FC_{s}\frac{A_{k,i}g_{k}}{U_{s}(T)}\exp(-E_{k}/k_{B}T)$$

where the subscripts *k* and *I* indicate the upper and lower energy levels of the transition line, respectively; *F* is an instrumental constant for fixed experimental conditions; *Cs* is the atomic or ionic number density of the specific element; *g*, *A*, and *U(T)*, are the statistical weight, transition probability and partition function at temperature *T*, respectively; *k_B_* and *E* represent the Boltzmann constant and excitation energy, respectively; and *I* is the spectrally integrated line intensity. When the plasma is in the local thermal equilibrium state, the plasma temperature can be approximated as a constant. Equation (1) can be simplified as,

(2) $$C_{S}=AI_{k,i}$$

The concentration of the element to be tested in the sample can be calculated according to Equation (2). However, due to the influence of the matrix effect, parameter *A* is difficult to determine experimentally. Moreover, when the concentration of the element increases, as self-absorption effect occurs, and the relationship between *C_s_* and *I_k,i_* can be expressed as,

(3) $$C_{S}=K_{b}{\left(I_{k,i}\right)}^{b}$$

where *K_b_* is the proportionality factor and *b* is the absorption coefficient. We simplified $I_{k,i}$, to *I*, and substituted it as a variable to support vector machine regression objective function,

(4) $$C_{S}=\Sigma_{i\in v}\partial_{i}k_{libs}(I_{i},I)+b$$

where *v* is the set of support vectors; $\partial_{i}$ is the Lagrange multiplier; $k_{libs}\;\left(I_{i},\;I\right)$ is the kernel function; and *b* is the constant.

A hybrid kernel function for LIBS is obtained by considering the relationship between the element concentration and spectrum line intensity (Equations (2) and (3)):

(5) $$k_{libs}(I_{i},I)=cII_{i}+(1-c)\exp(\frac{-∥I-I_{i}∥}{2g^{2}})$$

The mixed kernel function consists of two parts; the former is a linear kernel function $cII_{i}$, and the latter is a radial basis kernel function. The support vector machine kernel function is often used to solve nonlinear mapping problems in data. A large number of experiments and data have shown that the radial basis kernel function has high fitting and prediction accuracy, so it is usually selected as a kernel function for research. The adjustment of the parameters in the SVR largely determines the regression effect. When the radial basis kernel function is selected as the kernel function, the penalty coefficient *c* and the kernel parameter *g* are mainly optimized in the quantitative model of the compound fertilizer element analysis [24].

### 2.4. Parameter Optimization Methods

In related research, SVR optimization methods have mainly been summarized as non-heuristic and heuristic. The traditional grid search method (GSM) and experimental method are non-heuristic, but the experimental method uses several experiments to compare parameters and determine the optimal ones. It is time-consuming and not easy to find the optimal parameters, so this research did not use the experimental method. 

In the GSM method, X.L. Liu et al. proposed that the parameters *c* and *g* should be divided into an equal grid within a certain spatial range [25]. Every grid node then represents a set of parameters. In the optimization process, the optimal parameters were found by gradual approximation of all the nodes in the grid, and the *c* and *g* parameters with the smallest regression mean square error were taken as the optimal parameters. A large number of experimental studies have shown that the parameters *c* and *g* have interval sensitivity. If the parameter optimization interval can first be roughly determined, then an accurate search can be performed to reduce unnecessary calculations. First, a large step size should be used to perform a rough search in a large range and to select a set of *c, g* values for the minimum regression mean square error. If multiple sets of *c* and *g* values correspond to the minimum regression mean square error in the parameter selection process, then the group of *c* and *g* values with the smallest parameter *c* should be selected as the best parameters. Too high a value of parameter *c* would lead to an over-learning state, that is, a state where the RMSEC is small, but that of the prediction set is large, and the generalization ability of the SVR is reduced. After finding the local optimal parameters, we selected a cell in the vicinity of this group parameters and used a small step size to perform the second, finer search to find the final optimal parameters. Chen P W et al. proposed a global probability search algorithm based on biological mechanisms such as natural selection and genetic variation [26]. As with other heuristic search methods, the evolutionary mechanisms of organisms are simulated during evolutionary computation, starting from a set of solutions and evaluating their performance. Hybridization and mutational gene manipulations are then performed to generate a group of next-generation solutions with better performance metrics, until the final search for the global optimal solution. The particle swarm optimization (PSO) algorithm is proposed by J. Kennedy and R.C. Eberhart et al. and based on the study of the predation behavior of birds [27]. The solution of each problem is regarded as a bird in the search space, denoted as particles. In each iteration, the particle will track two “extreme values” to update itself; one is the optimal solution found by the particle itself, and the other is the current optimal solution found by the entire population. This extreme value is the global extreme.

In 1999, Suyken et al. added the squared error term to the standard SVR objective function and proposed the LS-SVR method [28]. In this method, the observed value is the sample value, and the theoretical value is the assumed fitting function. The fitting function model is then obtained when the objective function is the smallest. We set the objective function as

(6) $$h_{\theta }(x_{1},x_{2},\ldots x_{n})=\theta_{0}+\theta_{1}x_{1}+\ldots +\theta_{n-1}x_{n-1}$$

and the loss function as,

(7) $$J(\theta )=\frac{1}{2}{\left(X\theta -Y\right)}^{T}(X\theta -Y)$$

After derivation and sorting out the parameters as,

(8) $$\theta ={\left(X^{T}X\right)}^{-1}X^{T}Y$$

It can be seen that the LS algorithm is simple and efficient. The constraint avoids the quadratic programming in the objective function and solves the problems of robustness, sparseness, and large-scale operation, which greatly shortens the optimization time. However, there are also some limitations: (1) when the inverse matrix of $X^{T}X$ does not exist, the LS algorithm is no longer applicable, and data processing needs to remove redundant features; (2) the fitting function must be a linear function, which must be converted before use; and (3) when the sample feature number N is large, it takes a lot of time to calculate the inverse matrix, and it may not be able to be calculated. In this study, the number of compound fertilizer samples was small, and the characteristic dimension of SVR was declining. Thus, the LS algorithm could be used for parameter optimization.

## 3. Results

### 3.1. Spectral Analysis

**Figure 2:**
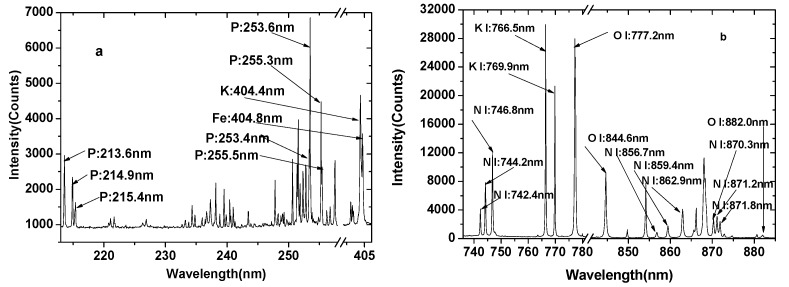
LIBS spectra of a compound fertilizer sample in the ranges of (**a**) 210–405 nm and (**b**) 740–890 nm.

Due to the existence of a large number of matrix element emission lines in the compound fertilizer, many characteristic lines interfered with each other. When selecting the characteristic line of an element, an unsaturated line with a high signal-to-noise ratio should be selected. According to the National Institute of Standards and Technology (NIST) database, the characteristic lines of elemental phosphorus are 213.5 nm, 214.9 nm, 215.4 nm, 253.4 nm, 253.6 nm, 255.3 nm, and 255.5 nm; the characteristic lines of elemental K are 404.7 nm, 766.5nm, and 769.9 nm; and the characteristic lines of elemental N are 742.4 nm, 744.2 nm, 746.8 nm, 856.7nm, 859.4 nm, 862.9 nm, 870.3 nm, 871.2 nm, and 871.8 nm. Figure 2 shows the spectrum of a compound fertilizer sample (Sample No.1) in the ranges of 210–405 nm and 740–890 nm. Although the two characteristic lines of P at 253.4 nm and 253.6 nm were strong, they were easily interfered with by the characteristic line of iron (Fe). In addition, the two lines at 255.3 nm and 255.5 nm were too close to distinguish. The characteristic lines at the wavelengths of 213.5 nm, 214.9 nm, and 215.4 nm were not interfered by other elements. It can be seen from Figure 2 that the characteristic lines of N were observed easily without any interference by other spectral lines, and the intensity at 746.8 nm was the strongest. The characteristic lines of elemental K in the compound fertilizer were not rich. Only three characteristic lines at 404.4 nm, 766.5 nm, and 769.9 nm were observed. The line at 404.4 nm was possibly interfered with by the characteristic line of Fe at 404.8 nm. However, the lines at 766.5 nm and 769.9 nm were too strong to be due to self-absorption. Elemental oxygen is also one of the main ingredients of compound fertilizer; the characteristic lines of O are 777.2 nm, 844.6 nm, and 882.0 nm.

### 3.2. Univariate Analysis

**Figure 3:**
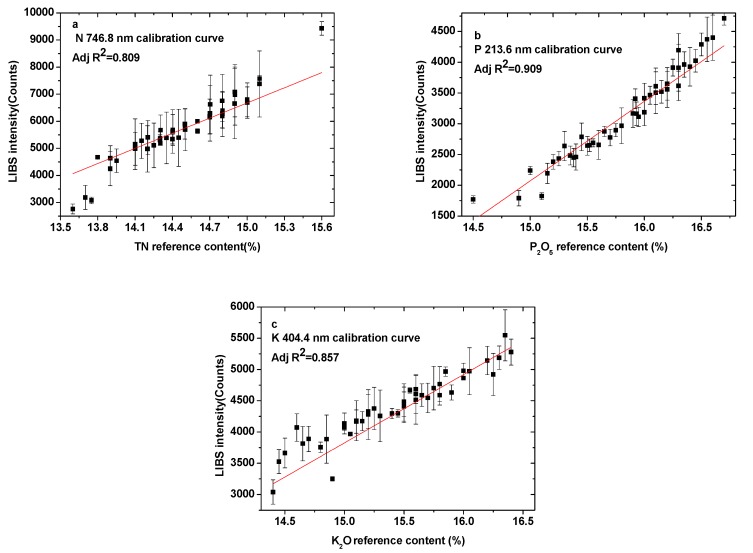
Calibration curves of the elemental spectral lines: (**a**) N: 746.8 nm, (**b**) P: 213.6 nm, (**c**) K: 404.4 nm.

Before modeling, the 58 fertilizer samples were divided into a calibration set (43 samples) and a prediction set (15 samples) using the Kennard–Stone (KS) method. The univariate calibration models were constructed using the line intensities (the height of Lorentz fits) of N at 746.8 nm, P at 213.6 nm, and K at 404.4 nm versus the corresponding contents. Figure 3a–c shows the calibration curves of N, P, and K elements, respectively. Figure 3a indicates the linear trend between the N line intensity and content, with a coefficient of correlation of 0.809. For P, the coefficient of correlation is 0.909, while it is 0.857 for K. For all three elements, the correlation coefficients cannot meet practical measurement needs and should be improved for further quantitative analysis.

### 3.3. SVR Analysis Models of Compound Fertilizer

SVR is a multivariate analytical technique which can make full use of spectral information and improve the accuracy of quantitative analysis by reducing the matrix effect. Calibration sets were used to construct the SVR model using the contents of N, P, and K elements and the LIBS spectral signal. These correlations were used to predict the contents of the prediction set. For each element, a proper spectral range was selected for modeling analysis in order to avoid over-fitting of the model. The reduced spectral ranges of 740–890 nm for N, 210–260 nm and 770–885 nm for P, and 400–410 nm and 770–885 nm for K were used to obtain the calibration model. MATLAB software was used for SVR model construction. The statistical parameters that determine the capacity of the regression model are the training time, the determination coefficients of the calibration set (R^2^_C_) and prediction set (R^2^_P_), and the RMSEC and RMSEP, which are given in this paper.

#### 3.3.1. Particle Swarm Optimization

**Figure 4:**
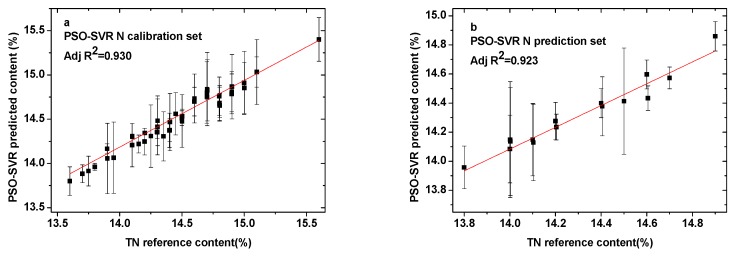

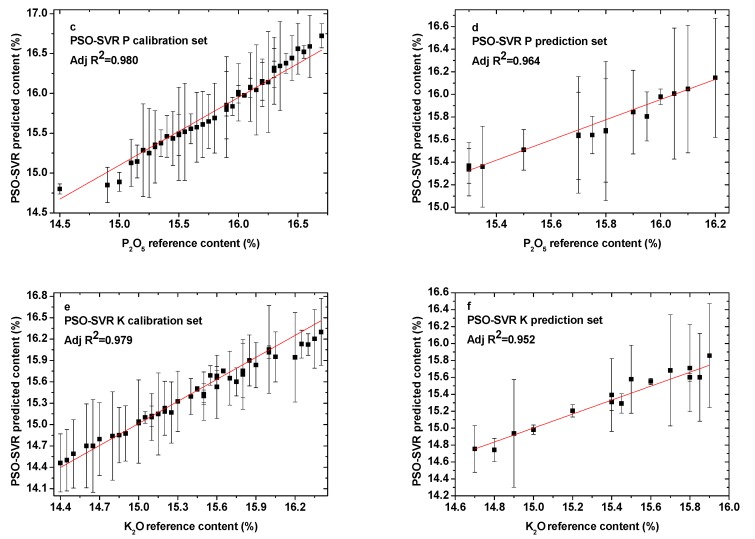
Comparison between PSO-SVR predicted content and reference content present in the (**a**) N calibration set; (**b**) N prediction set; (**c**) P calibration set; (**d**) P prediction set; (**e**) K calibration set; and (**f**) K prediction set.

Figure 4a–f shows the calibration and prediction results of SVR models using the PSO algorithm for N, P, and K elements, respectively. All of the parameters for both the calibration and prediction sets are presented in Table 2. The training times for N, P, and K were 2.98 s, 3.31 s, and 4.32 s, respectively. The determination coefficients for the calibration sets (R^2^_C_) were 0.930 for N, 0.980 for P, and 0.979 for K. Those for the prediction sets (R^2^_P_) were 0.923, 0.964, and 0.952. Meanwhile, for N, P, and K, respectively, the values of the RMSEC were 0.0996, 0.0701, and 0.0894, and those of the RMSEP were 0.0952, 0.0677, and 0.0921. The PSO-SVR optimization data showed that there was little difference between the three optimization times. The correlation coefficient between the N element calibration set and the prediction set was small and the error was large. The determination coefficients R^2^_C_ and R^2^_P_ for N indicated that the correlation needed to be improved. Meanwhile, the RMSEC and RMSEP for elements N and K were large. 

#### 3.3.2. Genetic Algorithm

**Figure 5:**
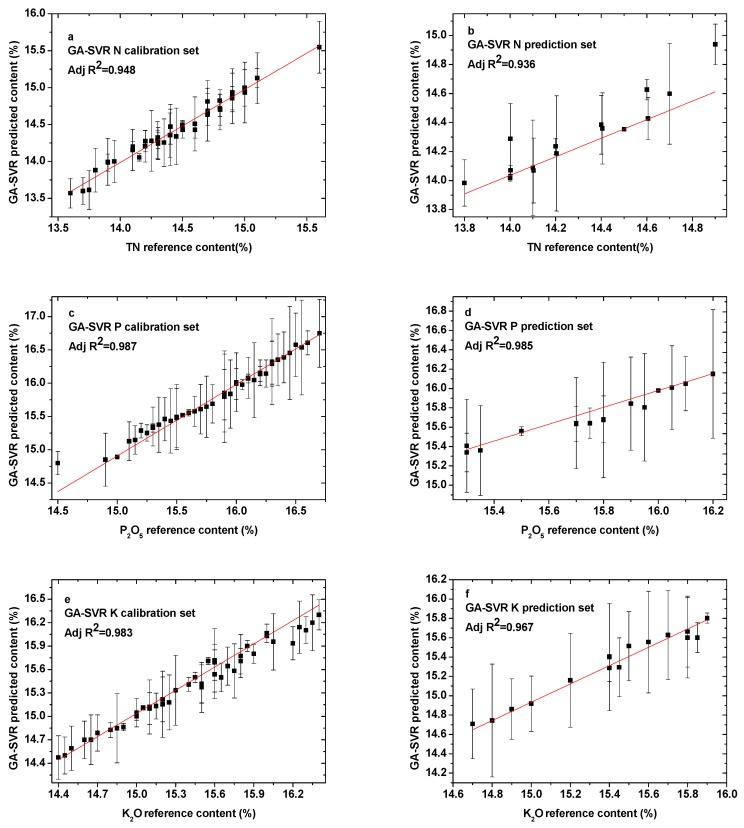
Comparison between GA-SVR predicted content and reference content present in the (**a**) N calibration set; (**b**) N prediction set; (**c**) P calibration set; (**d**) P prediction set; (**e**) K calibration set; and (**f**) K prediction set.

The optimal calibration and prediction results of N, P, and K elements based on parameters obtained using the genetic algorithm are shown in Figure 5a–f. Table 3 presents the detailed parameter results. The training times increased dramatically for all three elements. They were 5.67 s, 5.09 s, and 12.37 s, for N, P, and K, respectively. The R^2^_C_ of N increased from 0.930 to 0.948, but the R^2^_P_ changed from 0.923 to 0.936. The values of RMSEC and RMSEP for N were reduced to 0.0688 and 0.0694, which was better than the PSO algorithm. However, for elements P and K elements, the parameter optimization results of PSO and GA were basically the same, with only the R^2^_P_ value of P increased, from 0.964 to 0.985. This indicates that GA and PSO had many similarities, but GA was less inefficient due to random variation.

#### 3.3.3. Grid Search Method

**Figure 6:**
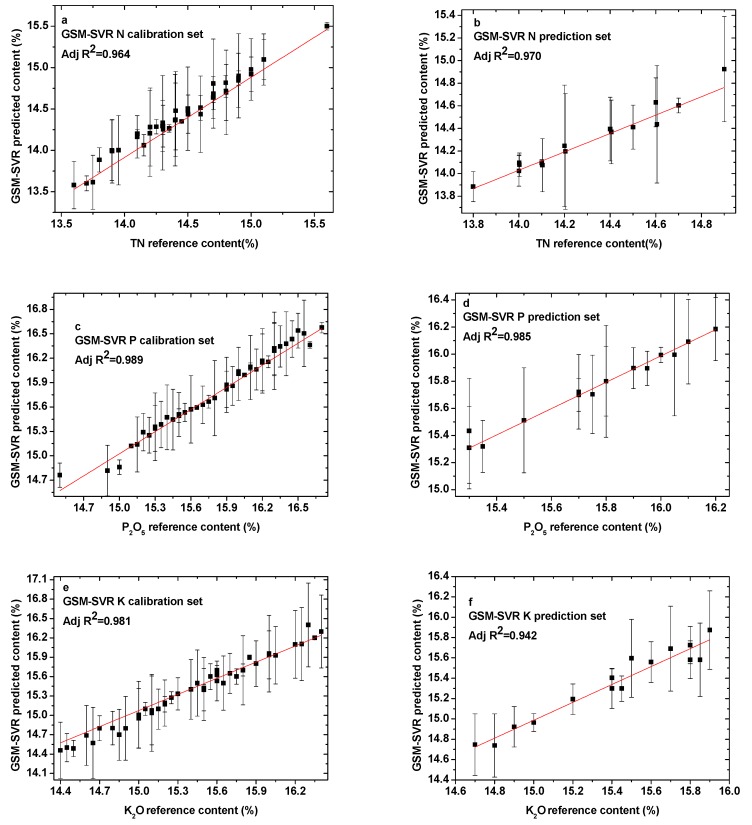
Comparison between GSM-SVR predicted content and reference content present in the (**a**) N calibration set; (**b**) N prediction set; (**c**) P calibration set; (**d**) P prediction set; (**e**) K calibration set; and (**f**) K prediction set.

A quantitative analysis SVR model of elements N, P, and K in the compound fertilizer was established based on parameter optimization by GSM. Figure 6a–f shows the calibration and prediction results of GSM parameter optimization for the elements N, P, and K. All of the results are presented in Table 4. For N, the training times, RMSEC, and RMSEP were almost identical to the results of the GA algorithm, but the R^2^_C_ and R^2^_P_ increased greatly. Among the three parameter optimization methods, for the P element, the results obtained by the GSM method were the best, and the training time was only 1.76 s. However, the best results for the K element were obtained by using the GA algorithm.

It can be seen that the quantitative analysis of the elements N, P, and K in compound fertilizer from the above three parameter optimization results was better than that by traditional methods [23], but it should be further improved.

#### 3.3.4. Least Squares

**Figure 7:**
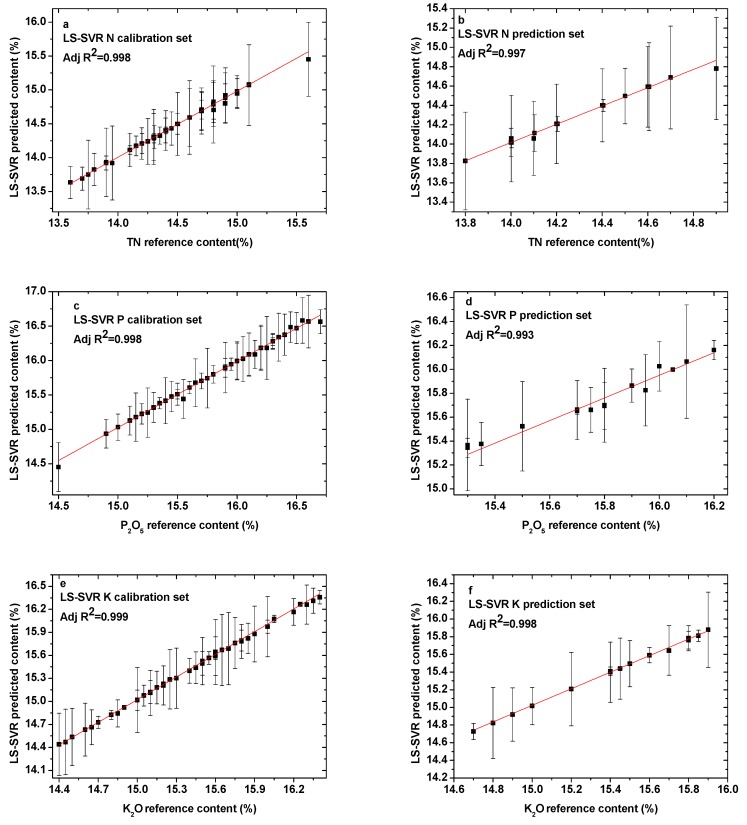
Comparison between the LS-SVR predicted content and reference content present in the (**a**) N calibration set; (**b**) N prediction set; (**c**) P calibration set; (**d**) P prediction set; (**e**) K calibration set; and (**f**) K prediction set.

The calibration and prediction results of the LS parameter optimization models for all analyzed elements are provided in Figure 7a–f. All of the specific parameter optimization results are stated in Table 5. It can be observed in Figure 6 that the calibration and prediction data points fitted well, indicating that the LS-SVR model had reliable prediction power for the quantitative analysis of compound fertilizer. In addition, the R^2^ values of the calibration and prediction sets for all elements were obviously improved. All the values of R^2^ were greater than 0.99. Meanwhile, for all elements, the values of RMSEC and RMSEP were significantly reduced. The values of RMSEC were reduced to 0.0240, 0.0258, and 0.0248 for elements N, P, and K, respectively, and the values of RMSEP were only 0.0218 for N, 0.0261 for P, and 0.0248 for K. The training time was significantly reduced; all the values of *t* were smaller than 0.3 s, which is more suitable for the rapid quantitative analysis of elements in compound fertilizer. Thus, it was demonstrated that the LS-SVR model can be developed to predict the content of unknown samples.

## 4. Conclusions

In summary, we demonstrated that LIBS coupling with the SVR method can provide a robust and accurate technology for the analysis of compound fertilizers. Four parameter optimization SVR models—the PSO model, the GA model, the GSM model, and the LS model were employed to quantitatively analyze elements N, P, and K in fertilizer. In general, the complex element composition of fertilizer causes difficulty for traditional calibration methods. For the conventional PSO, GA, and GSM parameter optimization methods, the determination coefficients for all three elements were greater than 0.92, and the root-mean-square errors were less than 0.101. However, the best parameter optimization model was the GSM method for N, GSM for P, and GA for K. A parameter optimization method suitable for quantitative analysis of all three elements was still needed. A LS-SVR model was then used to establish a quantitative analysis model for the three elements. From the results of the LS-SVR model, calibration and prediction models were obtained for the three elements with determination coefficients close to 1. For elements N, P, and K, respectively, the values of RMSEC were 0.0240, 0.0258, and 0.0239, and those of RMSEP were 0.0218, 0.0261, and 0.0248. After considering the evaluation indicators of the model comprehensively, the LS-SVR model is regarded as the most suitable for quantitative analysis of the three elements, with robust and satisfactory modeling performance. This model could therefore provide a basis for real-time analysis of N, P, and K elements in compound fertilizers. Furthermore, methods for improving the accuracy of the LIBS technique in the rapid detection of compound fertilizer on production lines will be the focus of future work.

## Figures and Tables

**Table 1:** Statistics of the effective constituents of compound fertilizer samples.

Properties	Total Nitrogen (TN/%)	P_2_O_5_ (%)	K_2_O (%)
Minimum value	13.60	14.50	14.40
Maximum value	15.60	16.70	16.40
Mean value	14.42	15.79	15.39
Standard deviation values	2.86	2.97	3.29

**Table 2:** The results of the particle swarm optimization–support vector regression (PSO-SVR) model for elements N, P, K.

Element	t/s	R^2^_C_	RMSEC	R^2^_P_	RMSEP
N	2.98	0.930	0.0996	0.923	0.0952
P	3.31	0.980	0.0701	0.964	0.0677
K	4.32	0.979	0.0894	0.952	0.0921

**Table 3:** The results of the genetic algorithm–support vector regression (GA-SVR) model for N, P, and K.

Element	t/s	R^2^_C_	RMSEC	R^2^_P_	RMSEP
N	5.67	0.948	0.0688	0.936	0.0694
P	5.09	0.987	0.0692	0.985	0.0680
K	12.37	0.983	0.0775	0.967	0.1007

**Table 4:** The results of grid search method–support vector regression (GSM-SVR) model for elements N, P, and K.

Element	t/s	R^2^_C_	RMSEC	R^2^_P_	RMSEP
N	4.89	0.964	0.0685	0.970	0.0712
P	1.76	0.989	0.0632	0.985	0.0576
K	4.21	0.981	0.0942	0.942	0.0969

**Table 5:** The results of the least squares–support vector regression (LS-SVR) model for elements N, P, and K.

Element	*t/s*	R^2^_C_	RMSEC	R^2^_P_	RMSEP
N	0.23	0.998	0.0240	0.997	0.0218
P	0.02	0.998	0.0258	0.993	0.0261
K	0.02	0.999	0.0239	0.998	0.0248
